# A new, mesophotic *Coryphopterus* goby (Teleostei, Gobiidae) from the southern Caribbean, with comments on relationships and depth distributions within the genus

**DOI:** 10.3897/zookeys.513.9998

**Published:** 2015-07-17

**Authors:** Carole C. Baldwin, D. Ross Robertson

**Affiliations:** 1 Department of Vertebrate Zoology, National Museum of Natural History, Smithsonian Institution, Washington, DC 20560; 2 Smithsonian Tropical Research Institute, Balboa, Republic of Panamá

**Keywords:** *Coryphopterus curasub*, *Coryphopterus dicrus*, submersible, Substation Curaçao, Deep Reef Observation Project (DROP), DNA barcoding, phylogeny

## Abstract

A new species of western Atlantic *Coryphopterus* is described from mesophotic depths off Curaçao, southern Caribbean. *Coryphopterus curasub* **sp. n**., is similar to *Coryphopterus dicrus* in, among other features, having two prominent pigment spots of roughly equal intensity on the pectoral-fin base, the pelvic fins fused to form a disk, and no pelvic frenum. The two species can be differentiated by body depth (shallower in *Coryphopterus curasub* at origin of dorsal fin and caudal peduncle); differences in the pigmentation on the head, trunk, and basicaudal region; and usually by total number of rays (spinous plus soft) in the second dorsal fin (10–11, usually 11, in *Coryphopterus curasub*, 10 in *Coryphopterus dicrus*). *Coryphopterus curasub* differs from other *Coryphopterus* species that have a prominent pigment spot on the lower portion of the pectoral-fin base (*Coryphopterus punctipectophorus* and *Coryphopterus venezuelae*) in, among other features, lacking a pelvic frenum. *Coryphopterus curasub* was collected between 70 and 80 m, the deepest depth range known for the genus. Collections of *Coryphopterus venezuelae* at depths of 65–69 m extend the depth range of that species by approximately 50 m. Mitochondrial cytochrome c oxidase subunit I (COI) data corroborate the recognition of *Coryphopterus curasub* as a distinct species but do not rigorously resolve its relationships within the genus. A revised key to the western Atlantic species of *Coryphopterus* is presented.

## Introduction

*Coryphopterus* gobies live in mostly shallow warm waters of the western Atlantic and eastern Pacific Oceans, dwelling on sand around coral and rocky reefs or hovering above or perching on reef structures. Twelve species are known from the western Atlantic and one from the eastern Pacific (Böhlke and Robins 1960, 1962; Thacker and Cole 2002; Victor 2007; Baldwin et al. 2009). Most *Coryphopterus* species inhabit depths < 40 m, but *Coryphopterus hyalinus* Böhlke & Robins has been recorded to 52 m; *Coryphopterus eidolon* Böhlke & Robins and *Coryphopterus thrix* Böhlke & Robins to 54 m; *Coryphopterus dicrus* Böhlke & Robins to 56 m; *Coryphopterus lipernes* Böhlke and Robins to 60 m; *Coryphopterus glaucofraenum* Gill to 61 m; and *Coryphopterus personatus* (Jordan & Thompson) to 70 m (Böhlke and Robins 1960, 1962; Thacker and Cole 2002; Feitoza et al. 2005, Robertson and Van Tassell 2015). Published depth data for *Coryphopterus glaucofraenum*, however, as well as that for *Coryphopterus tortugae* (Jordan) (5–32 m) and *Coryphopterus venezuelae* Cervigón (1–20 m), must be interpreted cautiously because of historical confusion about the taxonomy of this group, as should those of *Coryphopterus hyalinus* and *Coryphopterus personatus* for the same reason (Baldwin et al. 2009). Recent submersible diving to 300 m off Curaçao in the southern Caribbean as part of the Smithsonian Institution’s Deep Reef Observation Project (DROP) resulted in the collection of three specimens of *Coryphopterus venezuelae* at 65–69 m and four specimens of an unidentified *Coryphopterus* at 70–80 m. We describe the specimens from 70–80 m as a new species and comment on its relationships within the genus.

Thacker and Cole (2002) investigated species relationships within *Coryphopterus* based on morphology and one mitochondrial gene (ND2). Their phylogeny suggests that *Coryphopterus* is restricted to the western Atlantic and eastern Pacific and that the Indo-Pacific genus *Fusigobius*, which Randall (1995) synonymized with *Coryphopterus*, is distinct. Based on study of the western Atlantic *Lophogobius cyprinoides*, Thacker and Cole (2002) and Thacker and Roje (2011) hypothesized that *Coryphopterus* is more closely related to *Lophogobius* than it is to *Fusigobius*. The eastern Pacific *Coryphopterus nicholsii* (Bean), which is sister to *Lophogobius* + *Coryphopterus* in the phylogenies of Thacker and Cole (2002) and Thacker and Roje (2011), has been re-relegated to the monotypic genus *Rhinogobiops* Hubbs (Thacker 2011, Van Tassell 2011).

A new western Atlantic species, *Coryphopterus kuna* Victor, was described in 2007 but not included in the molecular phylogeny of Thacker and Roje (2011). Further, neither *Coryphopterus tortugae* nor *Coryphopterus venezuelae* was included in previous phylogenetic work, even though both appear to be valid *Coryphopterus* species. Although Longley and Hildebrand (1941) and Böhlke and Robins (1960) considered *Coryphopterus tortugae* (Jordan) to be a synonym of *Coryphopterus glaucofraenum*, Garzón-Ferreira and Acero (1990) redescribed it as valid. Victor (2008) concurred and also described a variant of *Coryphopterus tortugae* as new species, *Coryphopterus bol*. Based on an integrative molecular and morphological analysis, Baldwin et al. (2009) also recognized *Coryphopterus tortugae* as valid but relegated *Coryphopterus bol* to the synonymy of *Coryphopterus venezuelae*. In addition to the new species described here, tissue samples of all known *Coryphopterus* species except the Gulf of Mexico species *Coryphopterus punctipectophorus* Springer are now available and were incorporated into the genetic analyses conducted in this study. As noted by Baldwin et al. (2009), Thacker and Cole’s (2002) DNA sequence from Belize previously thought to be from *Coryphopterus punctipectophorus* (GenBank Accession No. AF391396) is actually from *Coryphopterus dicrus*.

## Materials and methods

Four specimens of the new species and three of *Coryphopterus venezuelae* were collected using Substation Curaçao’s (http://www.substation-Curacao.com) manned submersible *Curasub*. The sub has two flexible, hydraulic arms, one of which is equipped with a quinaldine-ejection system and the other with a suction hose. Anesthetized fish specimens were captured with the suction hose, which empties into a vented plexiglass cylinder attached to the outside of the sub. At the surface, the specimens were photographed, tissue sampled, and preserved. Preserved specimens were later photographed to document preserved pigment pattern and X-rayed with a digital radiography system. Counts and measurements follow Randall (2001). Format for dorsal-fin formula follows Birdsong et al. (1988). Head pore terminology follows Akihito et al. (1988). Measurements were made weeks to months after preservation and were taken to the nearest 0.1 mm with digital calipers or an ocular micrometer fitted into a Zeiss stereomicroscope.

Tissue samples for DNA Barcoding were stored in saturated salt-DMSO (dimethyl sulfoxide) buffer (Seutin et al. 1991). DNA extraction, PCR, sequencing cytochrome c oxidase subunit I (COI), and editing COI sequences were performed as outlined by Weigt et al. (2012a). A neighbor-joining tree (Saitou and Nei 1987) was generated using PAUP*4.1 (Swofford 2002) on an analysis of Kimura two-parameter distances (Kimura 1980). The neighbor-joining analysis reveals genetic distances in COI among individuals and clusters them into genetically distinct lineages, which, in teleost fishes, correspond well with species (e.g. Baldwin and Weigt 2012, Weigt et al. 2012b). Interspecific phylogenetic relationships were hypothesized for *Coryphopterus* based on maximum parsimony analysis of the COI sequences using heuristic searches in PAUP*4.1 (Swofford 2002). Characters were equally weighted and left unordered. The resulting equally parsimonious trees were summarized using the strict consensus method, and nodal support was estimated from 1,000 replicates of the bootstrap, utilizing random addition sequence and TBR branch swapping (Swofford 2002). The outgroup for the neighbor-joining analysis was a species of *Fusigobius*, a basal genus in the crested goby group that includes *Coryphopterus*, *Lophogobius*, and *Rhinogobiops* (Thacker and Roje 2011). Outgroups for the parsimony analysis were *Fusigobius* and *Rhinogobiops*.

GenSeq nomenclature (Chakrabarty et al. 2013) and GenBank accession numbers for DNA sequences derived in this study are presented along with museum catalog numbers for voucher specimens in the Appendix. GenBank accession numbers for *Coryphopterus* sequences included in the analyses that were published by Baldwin et al. (2009) are GQ367306–GQ367475, and those for *Lophogobius cyprinoides* sequences published by Weigt et al. (2012b) are JQ840574.1 and JQ842196.1. GenBank accession numbers for *Fusigobius duospilus* and *Rhinogobiops nicholsii* are JX462852 and HQ909488, respectively.

## Results

### Coryphopterus curasub sp. n.

Taxon classificationAnimaliaGobiiformesGobiidae

http://zoobank.org/5C60A7B0-58D9-4896-81EF-A0D1DD28873D

**Figure 1.**
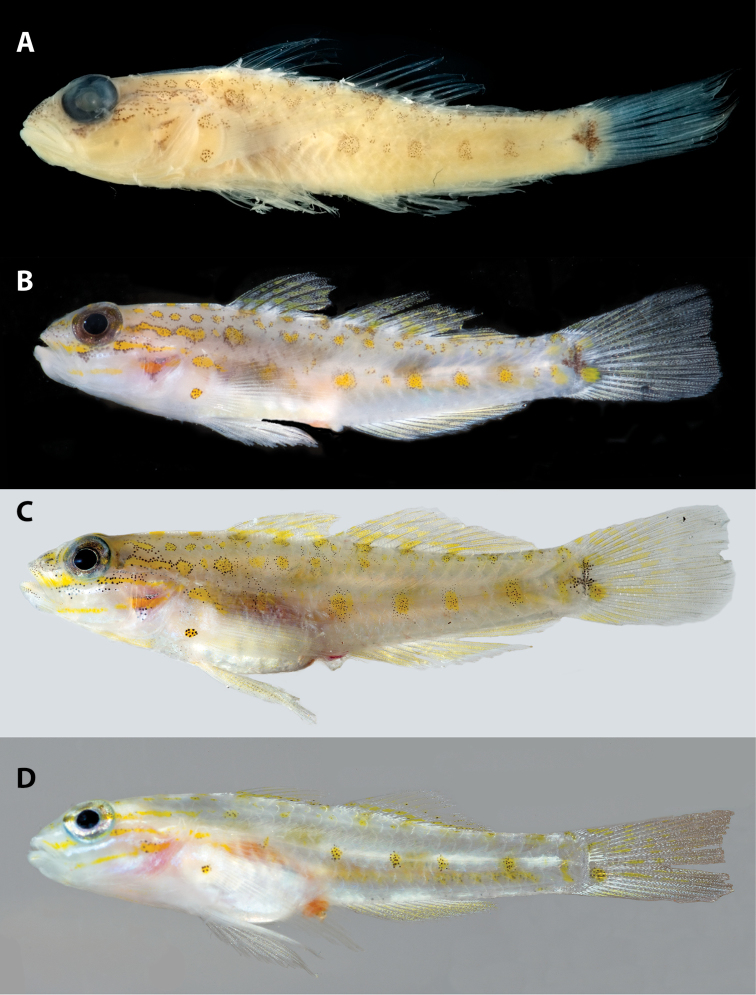
*Coryphopterus curasub* sp. n., type specimens: **A, B** USNM 406373, holotype, Smithsonian DNA number CUR 11373, 33.3 mm SL, female – after preservation (**A**) and before preservation (**B**) **C** USNM 431328, Paratype, CUR 14003, 31.0 mm SL, male, before preservation **D** USNM 430019, Paratype, CUR 13303, 17.5 mm SL, immature, before preservation and clearing and staining. Note that the dark color on the posterior portion of the caudal fin is an artifact of flash photography and does not reflect the existence of dark pigment. Photos by Ian Silver-Gorges (**A**) and D. R. Robertson and C. C. Baldwin (**B–D**).

**Figure 2.**
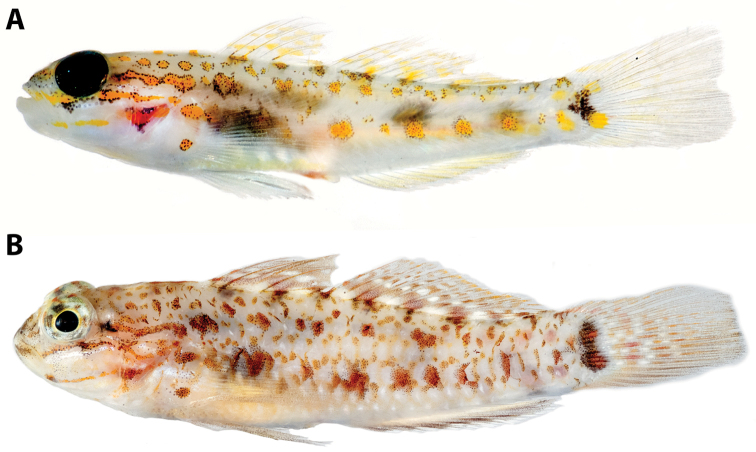
Comparison of **A** *Coryphopterus curasub* sp. n., holotype, USNM 406373, 33.3 mm SL, and its most similar congener **B** *Coryphopterus dicrus*, USNM 413296, 30 mm SL. Note the differences in the shape of the basicaudal pigment marking (with distinct anterior projection in *Coryphopterus curasub*), body depth (shallower in *Coryphopterus curasub*), head pigment (absence of a distinct blotch of black pigment immediately posterior to the orbit and presence of a black triangle of pigment beneath the anteroventral portion of orbit in *Coryphopterus curasub* (present and absent, respectively, in *Coryphopterus dicrus*), and trunk pigment (blotches predominantly yellow with few melanophores interspersed among them in *Coryphopterus curasub* vs. blotches predominantly orange/rust with numerous melanophores interspersed among them in *Coryphopterus dicrus*).

 Yellow-spotted sand goby Figs 1 , 2

#### Type locality.

Curaçao, southern Caribbean

#### Holotype.

USNM 406373, Smithsonian DNA number CUR 11373, 33.3 mm SL, female, *Curasub* submersible, sta. 11-05, southern Caribbean, Curaçao, east of downline off Substation Curaçao dock, near 12°05.069'N, 68°53.886'W, 80 m, quinaldine, 30 May 2011, D. R. Robertson, B. Brandt, A Schrier, K. Stewart.

#### Paratypes.

USNM 430037, CUR 13302, 30.0 mm SL, male, *Curasub* submersible, sta. 13-29, southern Caribbean, Curaçao, east of downline off Substation Curaçao dock, near 12°05.069'N, 68°53.886'W, 70–72 m, quinaldine, 30 October 2013, C. C. Baldwin, D. R. Robertson, B. Brandt, C. Castillo, L. Ybarrondo. USNM 431328, CUR 14003, 31.0 mm SL, male, *Curasub* submersible, sta. 14-01, southern Caribbean, Curaçao, east of downline off Substation Curaçao dock, near 12°05.069'N, 68°53.886'W, 73 m, quinaldine, 17 March 2014, C. C. Baldwin, D. R. Robertson, B. Brandt, C. Castillo, H. Reichardt. USNM 430019, CUR 13303, 17.5 mm SL, immature (same collection locality as USNM 430037), cleared and stained.

#### Generic assignment.

The combination of six spines in the first dorsal fin, fewer than 20 rays in the second dorsal fin, pelvic fin with one spine and five soft rays, head pores present, no free pectoral-fin rays, no scales on top of head, and no prominent crest on top of head anteriorly from first dorsal fin support the placement of *Coryphopterus curasub* in the genus *Coryphopterus* (Murdy 2002).

#### Diagnosis.

A species of *Coryphopterus* distinguishable from its congeners by the following combination of characters: total second dorsal-fin rays (spinous plus soft) 10-11, usually 11; total anal-fin rays (spinous plus soft) 10; pectoral-fin rays 19-20; pelvic fins united; no pelvic frenum; pectoral-fin base with two prominent dark spots (yellow with dark spotting in life) of roughly equal intensity, one on dorsal portion of fin base and one on ventral part; no distinct black blotch behind orbit above opercle; no dark triangular blotch immediately behind middle of orbit; blotches of pigment on trunk mostly yellow; few melanophores and yellow dots interspersed among yellow blotches of pigment on trunk; no black ring of pigment surrounding anus; dark triangular blotch variously developed beneath anteroventral corner of orbit; basicaudal blotch cross-shaped, with prominent anterior projection; and two yellow/orange blotches on base of caudal fin situated immediately behind basicaudal blotch.

#### Description.

Dorsal-fin rays VI + I, 9-10 (9 in one paratype, 10 in other specimens), total second dorsal-fin rays 10 or 11 (10 in one paratype, 11 in other specimens); anal-fin rays I, 9; all soft dorsal- and anal-fin rays branched. Pectoral-fin rays 19, 19 (paratypes) or 20, 20 (holotype); all pectoral rays branched except splint-like uppermost and lowermost rays. Pelvic-fin rays I, 5; all soft rays branched; fins united, no frenum. Total caudal-fin rays (including procurrent rays) 30 (holotype) or 31 (cleared and stained paratype; can’t assess number from radiographs of other paratypes); segmented caudal rays17; branched caudal rays12 (6+6); unbranched caudal rays 18 in holotype (9+9), 19 (10+9) in cleared and stained paratype. Dorsal-fin formula 3-22110. Vertebrae 10 precaudal +16 caudal. Epineurals 10 pairs. Ribs on vertebrae 3-10. Anal-fin pterygiophores anterior to first haemal spine 2. Gill rakers on first arch, including rudiments, 2 + 8 (holotype and adult paratypes), 0 + 8 in cleared and stained juvenile paratype. Branchiostegal rays 5. Numerous scales abraded and missing on all specimens, one paratype with approximately 22 scales in longitudinal series, 6 scales between origin of second dorsal fin obliquely downward to anal fin, and approximately 17 circum-peduncular scales.

Measurements of holotype in parentheses following extremes for holotype plus two adult paratypes. Juvenile paratype bent and not measured prior to clearing and staining. Body elongate, maximum depth from base of dorsal-fin spines 17–19% SL (17); body compressed, greatest width just posterior to gill opening 14–15% SL (14); head length 30–32% SL (32); snout length (to fleshy edge of orbit) 6.0–7.1% SL (6.0); greatest fleshy orbit diameter 9.3–10% SL (9.3); least fleshy interorbital 1.3–3.3% SL (3.3); caudal-peduncle length 23–24% SL (23); least caudal-peduncle depth 11–12% SL (11); length of dorsal-fin base 39–46% SL (46); first dorsal spine 14–16% SL (16); second dorsal spine 15–18% SL (17); third and longest dorsal spine 16–20% SL (18); sixth and shortest dorsal spine 7.0–8.4% SL (8.4); seventh dorsal spine (first element of second dorsal fin) 14–16% SL (16); last dorsal soft ray 16–17% SL (16); anal-fin spine 8.7–11% SL (11); last anal soft ray 20–22% SL (20); pectoral fin 32–36% SL (36), fin reaching vertical through second anal-fin soft ray, longest pectoral rays the 11^th^-14^th^ rays from top of fin; pelvic fin 24–32% SL (27), fin reaching origin of anal fin or terminating slightly before origin, longest pelvic ray the fourth.

Trunk, belly, and pre-pelvic region scaled, head and predorsal region naked; scales ctenoid except on pre-pelvic region, where they are cycloid. Upper jaw with several rows of small conical teeth, outermost teeth largest but smaller than outermost dentary teeth; dentary with outer row of fairly large conical teeth and several inner rows of smaller teeth; innermost teeth intermediate in size between teeth of outermost row and those adjacent to it. No teeth on vomer, palatines, or pterygoids. Anterior nare opening on short tube; posterior nare a simple opening. Head pores prominent: nasal pore, anterior interorbital pore, posterior interorbital pore, infraorbital pore, postorbital pore, pore at each end of lateral sensory canal, pore at each end of posterior lateral canal, and three preopercular pores (pores B’, C and D [both single], E, F, G, H’, K’, L’, M’, N, O’). A very low, thin ridge of tissue extending from just posterior to interobital region to base of first dorsal fin.

When photographed against a light background (Fig. 2A), the following color pattern visible in holotype. TRUNK: ground color white, several irregular horizontal rows of yellow/orange irregular-shaped blotches, most blotches bordered by and peppered with small black melanophores; uppermost row comprising approximately 11 blotches distributed along dorsal body margin from head (with two-three blotches) to caudal peduncle; second row shortest, comprising approximately eight blotches and extending from just posterior to posterodorsal margin of orbit to vertical through third or fourth dorsal-fin spine; third row comprising approximately 15 blotches and extending from middle of posterior margin of orbit to caudal peduncle—anterior blotches of this row united to form irregular stripe; lowermost row comprising five prominent blotches that extend from vertical through anterior origin of second dorsal fin to posterior portion of caudal peduncle and several less conspicuous blotches anterior of and within this series; this row continuing anteriorly onto pectoral-fin base and head as an irregular yellow stripe that passes along ventral margin of orbit and terminates on posterior end of upper jaw; yellow blotches on anterior portion of trunk and head better defined by peripheral melanophores than those on posterior portion of trunk; scattered small spots of yellow/black pigment interspersed among blotches in most rows. HEAD: head pigment also including short yellow/black stripe on snout; yellow stripe on ventral portion of head from posterior end of lower jaw to preopercle; triangle of black pigment beneath anteroventral margin of orbit; scattered black dots on upper lip, on snout, and beneath eye; two small black blotches of pigment on operculum; some whitish pigment extending posterodorsally from dark triangle beneath orbit; pupil black, iris brown; ventral portions of head and trunk mostly white except for streak of yellow pigment beneath opercular opening. CAUDAL PEDUNCLE: dark cross-shaped basicaudal blotch present on central portion of caudal peduncle and caudal-fin base, the anterior projection of cross prominent; two yellow spots bordering dorsal and ventral ends of blotch posteriorly and extending onto bases of several caudal-fin rays. FINS: first dorsal fin translucent, with three irregular yellow stripes; second dorsal fin with three or four irregular yellow stripes; anal fin with broad stripe of pale yellow pigment on middle of fin; caudal fin with blotches of yellow pigment forming an arc on basal portion of fin, some of this pigment extending distally along caudal rays as barely noticeable yellow streaks; pectoral and pelvic fins mostly clear; yellow/black blotch on dorsal portion of pectoral-fin base extending onto basal portion of dorsalmost pectoral rays; lower portion of pectoral-fin base with well-defined, round, yellow/orange blotch with dark dots. When photographed against a black background (Fig. 1B), numerous small, white, round to oblong spots visible on membranes of all fins; distal margin of anal fin with pale blue stripe.

Male paratypes (Fig. 1C) with similar coloration except black triangle of pigment beneath anteroventral corner of eye less conspicuous; melanophores present on lower jaw; iris mottled whitish/bronze; pectoral fin with pale yellow pigment; and pelvic fin with black-spotted yellow patches. IMMATURE SPECIMEN (Fig. 1D) paler but with most pigment described above developing; diagnostic cross-shaped marking on caudal peduncle not formed, and only lower black-spotted yellow blotch on pectoral-fin base well formed; most prominent pigment comprising yellow stripes on head, five black-spotted yellow blotches on trunk in lowermost row, and black-spotted yellow blotch in line with this row on base of caudal fin.

Color of holotype in alcohol (Fig. 1A). Ground color of head and trunk light tan, overlain by assorted dark circles, stripes, and irregular markings. Scattered melanophores and blotches present along base of spinous dorsal fin and on dorsal portion of trunk. Most prominent trunk pigment located just ventral to lateral midline as a row of six mostly circular blotches of roughly equal size except the second from anterior and last, which are small relative to the others; this row of pigment markings originating at a vertical through second element of second dorsal fin and terminating on caudal peduncle. Head with several circular blotches in row posterior to posterodorsal portion of orbit; irregular stripe of pigment extending posteriorly from middle of orbit; irregular stripe-like mark extending posteriorly from posteroventral portion of orbit; scattered melanophores on snout, lacrimal, and upper jaw; dark triangle of pigment beneath anteroventral corner of orbit; and two irregular streaks of pigment on ventral portion of operculum. Dark portion of basicaudal blotch as described above, but no melanophores present on remainder of caudal fin or on anal and pelvic fins. First dorsal fin with small bits of dark pigment on membranes of second, third and fifth spines. Pectoral-fin base with one dorsal and one ventral circular blotches, the former extending as short series of melanophores posteriorly onto bases of dorsal rays of fin.

#### Distribution.

Known from 70–80 m off Curaçao, southern Caribbean.

#### Habitat.

Notes recorded during the submersible dive on which the 33.0 mm SL paratype (USNM 431328) was collected indicate that it occurred on sand with rubble patches on a 45°slope.

#### Etymology.

Named for the manned submersible *Curasub*, which is owned and operated by Substation Curaçao, in recognition of the contributions of this vehicle to increasing our knowledge of the Caribbean deep-reef fish fauna.

#### Common name.

“Yellow-spotted sand goby” refers to the yellow spots on the trunk and the collection habitat.

#### Morphological comparisons.

*Coryphopterus curasub* is most similar to *Coryphopterus dicrus* (Fig. 2) and keys to that species in the most recent dichotomous key to western Atlantic *Coryphopterus* (Baldwin et al. 2009). They share the presence of two dark circular markings on the pectoral-fin base that are of roughly equal intensity (except in the juvenile *Coryphopterus curasub*, in which only the lower spot is prominent), the absence of a distinct black blotch or triangle of pigment behind the eye above the opercle in adults, the presence of a united pelvic fin in which the fourth rays are longer than the fifth, and the absence of a pelvic frenum. They usually differ in total number of rays (spinous plus soft) in the second dorsal fin (10–11, usually 11, in *Coryphopterus curasub*, 10 in *Coryphopterus dicrus*); absence of a dark triangular blotch immediately posterior to the orbit in *Coryphopterus curasub* (present in *Coryphopterus dicrus*); presence of a dark triangular blotch beneath the anteroventral portion of the orbit in the largest specimen (33.3 mm SL holotype) of *Coryphopterus curasub* (absent in similarly large specimens of *Coryphopterus dicrus*); blotches of pigment on the trunk typically yellow in *Coryphopterus curasub*, orange to rusty brown in *Coryphopterus dicrus*; few yellow spots with tiny melanophores among the yellow blotches of pigment on the trunk in *Coryphopterus curasub* vs. many rusty spots with tiny melanophores between the rusty brown blotches in *Coryphopterus dicrus*; configuration of the basicaudal blotch (a cross-shaped blotch with a distinct anterior projection in *Coryphopterus curasub* vs. a dumbbell-shaped bar in *Coryphopterus dicrus*); the two yellow/orange blotches on the base of the caudal fin situated immediately behind the basicaudal blotch in *Coryphopterus curasub* vs. superimposed on and contributing to the upper and lower heads of the basicaudal bar of *Coryphopterus dicrus*; maximum body depth from base of spinous dorsal fin (17–19% SL in *Coryphopterus curasub*, 20–26% SL in *Coryphopterus dicrus* – Böhlke and Robins 1960); least depth of caudal peduncle (11–12% SL in *Coryphopterus curasub*, 13–15% SL in *Coryphopterus dicrus* – Böhlke and Robins 1960). *Coryphopterus curasub* differs from all other western Atlantic *Coryphopterus* (*Coryphopterus alloides* Böhlke & Robins, *Coryphopterus eidolon*, *Coryphopterus glaucofraenum*, *Coryphopterus hyalinus*, *Coryphopterus kuna*, *Coryphopterus lipernes*, *Coryphopterus personatus*, *Coryphopterus punctipectophorus*, *Coryphopterus thrix*, *Coryphopterus tortugae*, and *Coryphopterus venezuelae*) in having two round dark marks on the pectoral-fin base that are of roughly equal intensity in adults. It further differs from *Coryphopterus hyalinus*, *Coryphopterus lipernes*, and *Coryphopterus personatus* in lacking a black ring around the anus; from those species and *Coryphopterus alloides* in having the pelvic fin united; from *Coryphopterus glaucofraenum*, *Coryphopterus tortugae*, and *Coryphopterus venezuelae* in lacking both a pelvic frenum and a distinct black blotch or triangle behind the eye above the opercle; and from *Coryphopterus kuna* in having 10 or 11 total second dorsal-fin rays, 10 total anal-fin rays, and 19–20 pectoral-fin rays (vs. 9, 9, and 15, respectively).

Of the 14 apomorphic morphological characters of *Coryphopterus* species tabulated by Thacker and Cole (2002) for inclusion in their phylogenetic analysis of the genus, *Coryphopterus curasub* has (character 1) no pelvic frenum, (3) the fifth (innermost) pelvic-fin ray shortened relative to the fourth, (4) a low ridge of tissue on top of the head, (8) orange or gold coloration on the body, and (10) three stripes of pigment on the head. The presence of a low ridge of tissue on the head characterizes all *Coryphopterus* species and is thus uninformative. Likewise, although Thacker and Cole (2002) scored most species as lacking orange or gold coloration, in a more thorough analysis of fresh color patterns in western Atlantic *Coryphopterus*, Baldwin et al. (2009) noted the presence of yellow/orange/gold pigment in all species. *Coryphopterus curasub* shares with *Coryphopterus dicrus*, *Coryphopterus alloides*, *Coryphopterus personatus*, *Coryphopterus hyalinus*, and *Coryphopterus lipernes* the absence of a pelvic frenum, with those taxa and *Coryphopterus eidolon* a shortened fifth pelvic-fin ray (relative to the fourth), and with *Coryphopterus eidolon*, *Coryphopterus thrix*, *Coryphopterus dicrus*, *Coryphopterus glaucofraenum*, *Coryphopterus tortugae*, *Coryphopterus venezuelae*, *Coryphopterus urospilus*, and *Coryphopterus punctipectophorus* the presence of three stripes of pigment on the head. In *Coryphopterus curasub*, the lowermost stripe (on the cheek) is yellow and lacks melanophores, which are present in the other species. The homology of the pigment stripes is thus questionable. Thacker and Cole (2002) list several apomorphic characters (11–13) related to basicaudal pigment, but the configuration of the basicaudal blotch in *Coryphopterus curasub* is unique among *Coryphopterus* species. Thacker and Cole’s (2002) 14^th^ character, the presence or absence of a pigment spot on the pectoral-fin base, insufficiently describes the variation in this character in *Coryphopterus*. Of the various configurations—no spots, one spot dorsally, one spot ventrally, two spots with upper spot more intense, two spots of roughly equal intensity—only *Coryphopterus curasub* and *Coryphopterus dicrus* have two spots of equal intensity among *Coryphopterus* species and outgroup taxa. In summary, of the potentially informative, putative apomorphic characters exhibited by *Coryphopterus curasub*, only *Coryphopterus dicrus* shares all of them.

#### Genetic comparisons.

**Figure 3.**
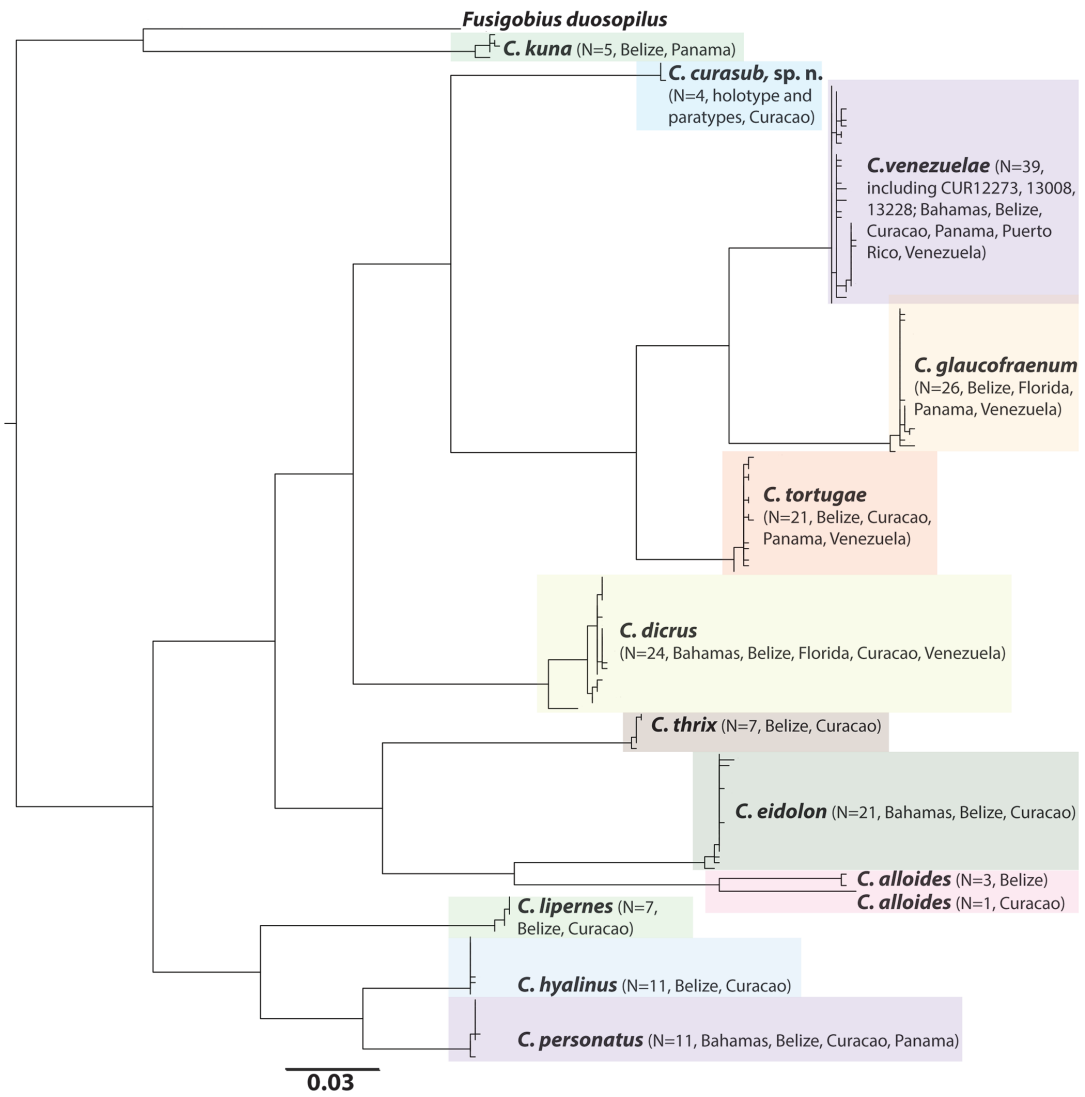
Neighbor-joining tree derived from COI sequences for western Atlantic species of *Coryphopterus*. The tree was rooted on *Fusigobius duospilus*. Divergence represented by scale bar = 3%. Note: *Coryphopterus punctipectophorus* from the Gulf of Mexico was not available for inclusion in this analysis.

**Figure 4.**
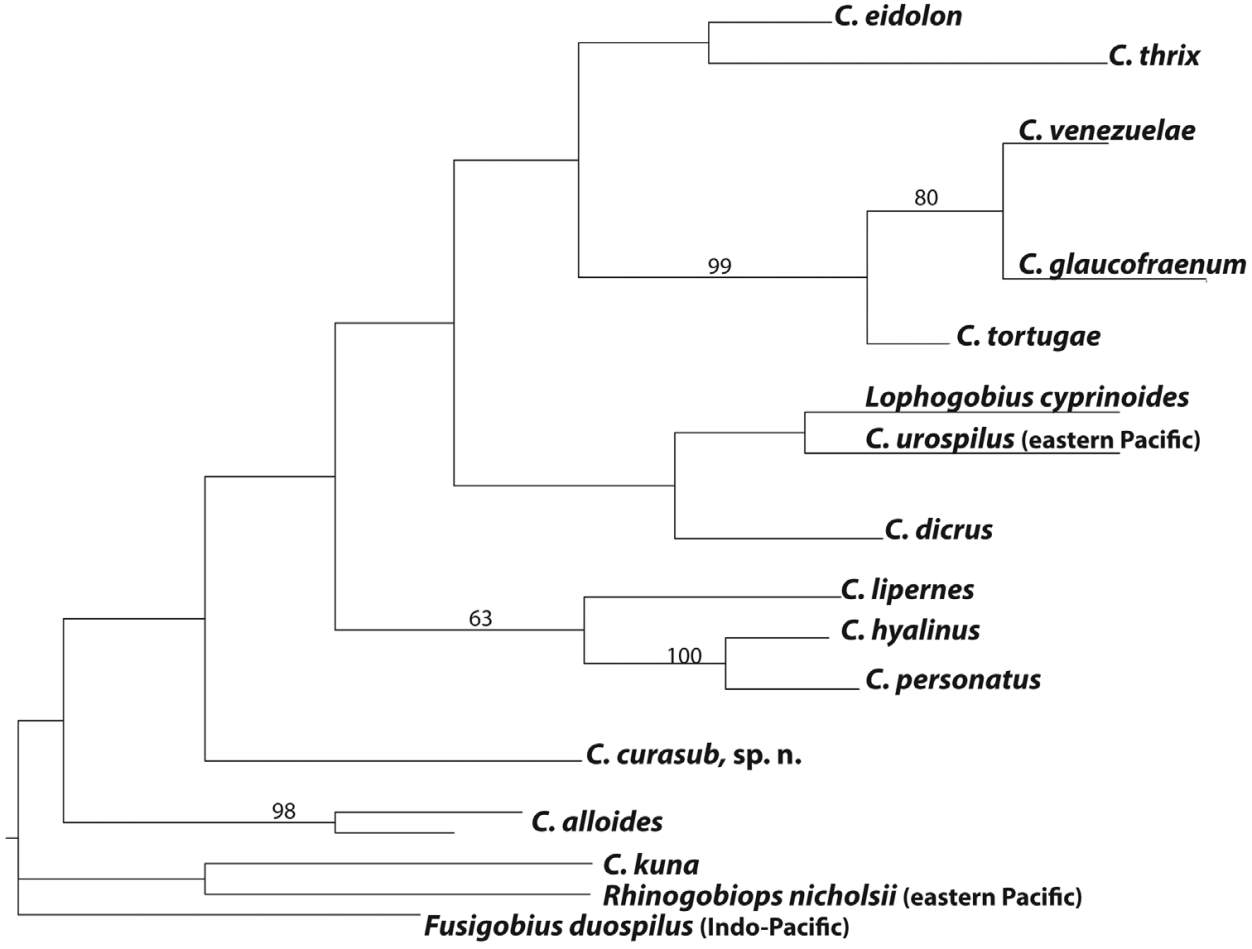
The strict consensus of a maximum parsimony analysis of the COI region of 42 individuals of *Coryphopterus* and *Lophogobius cyprinoides*. *Fusigobius duospilus* and *Rhinogobiops nicholsii* were outgroups in the analysis. Numbers above branches represent bootstrap support values > 50. Note: *Coryphopterus punctipectophorus* from the Gulf of Mexico was not available for inclusion in this analysis.

COI sequences derived from tissue samples from the four type specimens of *Coryphopterus curasub* and three specimens of *Coryphopterus venezuelae* collected by submersible as part of this study (Appendix) were combined with 173 previously published COI sequences for western Atlantic *Coryphopterus* (Baldwin et al. 2009, Weigt et al. 2012b) in a neighbor-joining analysis (Fig. 3). Intraspecific divergence in COI for *Coryphopterus curasub* was 0.1% as compared to 17–23% interspecific divergence between *Coryphopterus curasub* and other western Atlantic *Coryphopterus* species, including *Coryphopterus dicrus* (18%, Table 1). Intraspecific divergences for all western Atlantic *Coryphopterus* species were < 1% except for *Coryphopterus alloides* (3.7%), likely reflecting, as suggested by Baldwin et al. (2009), a cryptic species that awaits investigation. Phylogenetic relationships within *Coryphopterus* were analyzed using a reduced COI dataset of western Atlantic *Coryphopterus* (43 ingroup sequences selected from the entire COI data set to maximize geographical coverage of each species), the eastern Pacific *Coryphopterus urospilus*, and the western Atlantic *Lophogobius cyprinoides*. A strict consensus of 24 trees resulting from a maximum parsimony analysis (Fig. 4) does not resolve the relationships of *Coryphopterus curasub* with confidence. A clade comprising *Coryphopterus venezuelae*, *Coryphopterus glaucofraenum*, and *Coryphopterus tortugae* is strongly supported (99%), as are clades comprising *Coryphopterus venezuelae* and *Coryphopterus glaucofraenum* (80%), the hovering species *Coryphopterus hyalinus* and *Coryphopterus personatus* (100%), and the planktivores *Coryphopterus lipernes* + *Coryphopterus hyalinus* + *Coryphopterus personatus* (63%). As noted by Thacker and Cole (2002) based on ND2 mitochondrial and morphological data, *Lophogobius* is closely related to *Coryphopterus*, here appearing in a poorly supported clade that also comprises *Coryphopterus dicrus* and the eastern Pacific *Coryphopterus urospilus*. Adding more loci to the genetic analysis as well as *Coryphopterus punctipectophorus* and the eastern- and Indo-Pacific species of *Lophogobius* may help resolve interspecific and generic relationships. The placement of *Coryphopterus kuna* outside of the western Atlantic *Coryphopterus* + *Lophogobius cyprinoides* clade warrants further morphological and molecular investigation.

**Table 1.** Average Kimura two–parameter distance summary for species of western Atlantic *Coryphopterus* based on cytochrome c oxidase I (COI) sequences of individuals represented in the neighbor–joining tree in Figure 3. Intraspecific averages are shown in bold.

	*curasub*	*dicrus*	*lipernes*	*hyalinus*	*personatus*	*tortugae*	*glaucofraenum*	*venezuelae*	*thrix*	*eidolon*	*alloides*	*kuna*
*Coryphopterus curasub* (n=4)	**0.10**											
*Coryphopterus dicrus* (n=24)	18.00	**0.60**										
*Coryphopterus lipernes* (n=7)	19.40	21.70	**0.10**									
*Coryphopterus hyalinus* (n=11)	18.00	19.60	14.90	**0.10**								
*Coryphopterus personatus* (n=11)	19.00	19.00	15.60	7.20	**0.10**							
*Coryphopterus tortugae* (n=21)	17.40	17.50	19.60	21.10	20.10	**0.20**						
*Coryphopterus glaucofraenum* (n=26)	17.80	20.60	20.70	21.70	21.50	12.10	**0.20**					
*Coryphopterus venezuelae* (n=39)	17.40	18.30	21.40	20.90	20.10	9.90	9.50	**0.40**				
*Coryphopterus thrix* (n=7)	22.90	21.30	21.90	21.10	19.70	19.00	21.20	19.60	**0.20**			
*Coryphopterus eidolon* (n=21)	22.90	19.40	25.20	19.20	18.00	19.70	12.10	18.90	19.60	**0.10**		
*Coryphopterus alloides* (n=4)	17.90	17.90	21.90	17.90	18.40	20.00	21.30	18.90	20.20	18.50	**3.70**	
*Coryphopterus kuna* (n=5)	22.70	24.90	26.40	23.20	25.70	25.50	27.90	26.00	24.90	25.80	23.60	**0.50**

#### Depth distributions.

**Figure 5.**
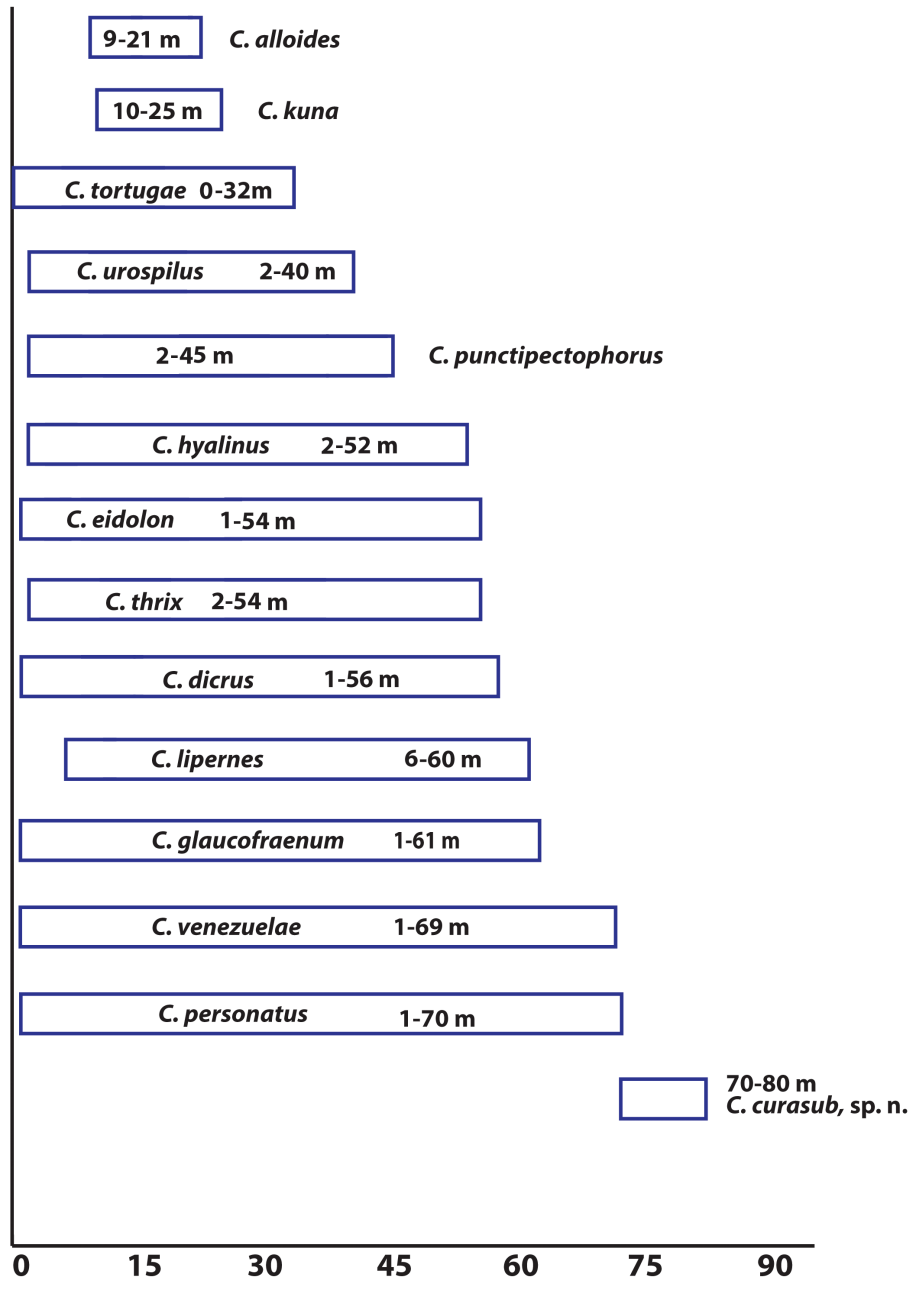
Depth ranges for *Coryphopterus* species. Data are from Böhlke and Robins (1960, 1962); Thacker and Cole (2002); Feitoza et al. (2005); Robertson and Van Tassell (2015); the Florida Museum of Natural History online fish catalog – http://specifyportal.flmnh.ufl.edu/fishes/; the Florida Fish and Wildlife Conservation Commission online catalog http://myfwc.com/research/saltwater/specimen-collections/sis/ichthyology/; the Smithsonian National Museum of Natural History online catalog – www.vertebrates.si.edu//search/fishes); and this study.

Depth ranges of *Coryphopterus* species are shown in Figure 5. *Coryphopterus curasub*, which is known from 70–80 m, is the only member of the genus that has a narrow depth range completely confined to mesophotic depths. *Coryphopterus venezuelae*, *Coryphopterus glaucofraenum*, *Coryphopterus dicrus*, *Coryphopterus eidolon*, *Coryphopterus thrix*, *Coryphopterus hyalinus*, *Coryphopterus lipernes*, and *Coryphopterus personatus* inhabit depths as deep as 52–70 m, but they have broad depth ranges that extend as shallow as 1–6 m. The only *Coryphopterus* species in addition to *Coryphopterus curasub* that we have collected using the *Curasub* submersible are *Coryphopterus hyalinus* – one specimen from 33 m, and *Coryphopterus venezuelae* – three specimens from 65–69 m. Prior to this study, *Coryphopterus venezuelae* was known from 1–20 m (Robertson and Van Tassell 2015), and thus our new collections of the species off Curacao extend its known range by nearly 50 m.

### Revised key to the Western Atlantic species of *Coryphopterus*

(Modified from Baldwin et al. 2009)

**Table:** 

1	Black ring surrounding anus; pelvic fins separate, frenum absent	**2**
–	No black ring around anus; pelvic fins separate or fused, frenum present or absent	**4**
2	One anterior interorbital pore	**3**
–	Two anterior interorbital pores	***Coryphopterus hyalinus***
3	Total second dorsal-fin rays (spinous plus soft) typically 11; total anal-fin rays (spinous plus soft) typically 11; in life, head with orange pigment, body translucent with several square- or rectangular-shaped orange blotches internally; preserved specimens lacking conspicuous postorbital stripes of melanophores but with dark “mask” around eye	***Coryphopterus personatus***
–	Total second dorsal-fin rays (spinous plus soft) typically 10; total anal-fin rays (spinous plus soft) typically 10; in life, head and body predominantly yellow, with blue-white stripes extending posteriorly from dorsal and ventral portions of orbit; a dusky internal stripe along posterior section of vertebral column; preserved specimens with postorbital stripes of melanophores and scattered spots over entire body	***Coryphopterus lipernes***
4	No distinct black blotch behind eye above opercle in adults; pigment mark above opercle, if present, no larger or darker than other marks behind eye; pelvic fins separate or fused, frenum present or absent (see Baldwin et al. 2009 for additional comments)	**5**
–	Distinct black blotch or triangle behind eye above opercle in adults, blotch usually larger and darker than other pigment in stripe behind eye; pelvic fins fused to form disc, frenum present (see Baldwin et al. 2009 for additional comments)	**11**
5	Total anal-fin rays (spinous plus soft) 8–9 (usually 9), pectoral-fin rays 15–17, pelvic fins separate or fused, frenum absent	**6**
–	Total anal-fin rays (spinous plus soft) 10–11, pectoral-fin rays 17– 20, pelvic fins fused, frenum present or absent	**7**
6	Total second dorsal-fin rays (spinous plus soft) 9; total anal-fin rays (spinous plus soft) 9; pectoral-fin rays 15; first dorsal fin with stripe of black pigment; in life, head and body with orange spots and blotches, sometimes a flag of dark pigment on 1st–3rd dorsal spines; pelvic fins fused to form a disc	***Coryphopterus kuna***
–	Total second dorsal-fin rays (spinous plus soft) 10; total anal-fin rays (spinous plus soft) 9 (rarely 8); pectoral-fin rays 16–17; black blotch or bar between 2nd and 3rd dorsal spines; in life, head and anterior portion of body mottled orange, posterior portion of body mottled yellow; pelvic fins separate	***Coryphopterus alloides***
7	Pectoral-fin base with two prominent dark spots of equal intensity, one dorsally and one ventrally; upper spot usually with swath of melanophores extending posteriorly onto pectoral-fin rays; sides of body freckled with scattered large and small blotches of melanophores (blotches associated with orange, rust, or yellow pigment in life); pelvic frenum absent	**8**
–	Pectoral-fin base with or without two prominent dark spots; if two spots present, upper spot more intense; sides of body with few dark markings (with few to many yellow spots in life) or with three rows of light markings (coral pink/orange in life); pelvic frenum present	**9**
8	Total second dorsal-fin rays (spinous plus soft) 10; a dark triangle immediately behind orbit; no dark triangle under front of orbit; basicaudal mark a vertical dumbbell that in life incorporates two large orange spots on the base of the caudal fin; maximum body depth beneath spinous dorsal fin 20–26% SL, least depth of caudal peduncle 13–15% SL, depth range 0–56 m	***Coryphopterus dicrus***
–	Total second dorsal-fin rays (spinous plus soft) 10 or 11, usually 11; no dark triangle behind orbit; dark triangle variously developed beneath front part of orbit; basicaudal blotch cross-shaped, the anterior horizontal projection prominent; in life, two large yellow spots on base of caudal fin just posterior to basicaudal blotch; maximum body depth beneath spinous dorsal fin 17–19% SL; least depth of caudal peduncle 9–12% SL; depth range 70–80 m	***Coryphopterus curasub* sp. n.**
9	Pectoral-fin base without prominent dark markings, but may have scattered melanophores; sides of body with few if any dark markings (with yellow to orange spots and stripes in life) except for several dark streaks internally along spinal cord and a thin dark basicaudal bar	***Coryphopterus eidolon***
–	Pectoral-fin base with prominent markings; sides of body with or without numerous dark markings	**10**
10	Pectoral-fin base with distinct, often large, pigment spot *dorsally*, spot usually dark above, diffuse below, often with dots trailing ventrally; ventral dots coalescing into a separate spot in some specimens (ventral spot, if present, less intense than dorsal spot); total second dorsal-fin rays (spinous plus soft) 9–10; second dorsal spine filamentous; in life, first dorsal fin without orange stripes	***Coryphopterus thrix***
–	Pectoral-fin base with prominent dark spot or blotch *ventrally*; total second dorsal-fin rays (spinous plus soft) 11; second dorsal spine not filamentous; in life, first dorsal fin with two broad orange stripes	***Coryphopterus punctipectophorus***
11	Body usually pale, pigment primarily comprising three rows of markings on side of body; lower row comprising small, mostly vertically elongate markings, some of which may be crescent shaped or some part of an X-shape but rarely well-defined X’s; height of any X-shaped markings considerably less than eye diameter; pigment mark above opercle usually a triangle, and basicaudal pigment usually a central bar	***Coryphopterus tortugae***
–	Body heavily pigmented or pale but no vertically elongate or crescent-shaped markings in ventral row of pigment on side of body; height of any X-shaped markings three-quarters of or equal to eye diameter; pigment mark above opercle triangular, rounded, or with two peaks; basicaudal pigment variable: two separate spots, a vertical dumbbell, a central bar, or a C-shaped marking	**12**
12	Pectoral-fin base with dark spot or rectangle *ventrally* (may be associated with bright yellow pigment in life); one or two bars or blotches sometimes present dorsally; three rows of dark markings on side of body, some in lower row large, X-shaped markings in heavily pigmented specimens, small, circular blotches in paler specimens; pigment mark above opercle triangular or round	***Coryphopterus venezuelae***
–	Pectoral-fin base rarely with prominent dark marking ventrally (may have one to three light to moderate concentrations of melanophores); body with three rows of dark marks, most of those in the lower row large and distinctively X-shaped; pigment mark above opercle usually with two well-defined peaks	***Coryphopterus glaucofraenum***

## Discussion and conclusions

Exploratory submersible diving to 300 m off Curaçao is resulting in the discovery of numerous new fish species, only a few of which have been described to date (Baldwin and Robertson 2013, 2014; Baldwin and Johnson 2014). In addition to the new *Coryphopterus* described here, numerous seven-spined gobies that represent undescribed species in the *Chriolepis*/*Psilotris*/*Varicus* group have been collected, as have several new species of *Lythrypnus*-like gobies and a putative new species of *Palatagobius*. A new genus and species of deep-reef goby, *Antilligobius nikkiae* Van Tassell, Tornabene, and Colin, was recently described from deep reefs at several localities in the Caribbean (including Curaçao) and Bahamas (Van Tassell et al. 2012). Deep-reef fish faunas in general have been poorly studied globally, and the recent new-species discoveries suggest that our knowledge of the deep-reef gobiid fauna in the southern Caribbean, and likely circumglobally, is far from complete. One question of interest is whether deep-reef species generally represent single offshoots of largely shallow-reef clades or form natural evolutionary groups. *Antilligobius nikkiae*, which inhabits depths of 73–150 m, appears to have its closest relative in shallower water than it inhabits. Rüber et al. (2003) hypothesized that *Antilligobius* belongs within the monophyletic *Microgobius* group of the tribe Gobiosomatini, specifically as the sister group of the monophyletic *Microgobius*. Tornabene et al. (2012) tabulated depth distributions for *Microgobius* species, which occur from < 1 m to at least 75 m, but most occur at depths < 20 m. The COI data analyzed in this study do not rigorously resolve relationships among *Coryphopterus* species, but morphological data suggest that *Coryphopterus curasub* may be most closely related to *Coryphopterus dicrus*. Depth ranges of those species (1–56 m for *Coryphopterus dicrus*, 70–80 m for *Coryphopterus curasub*) do not overlap, suggesting that if they are sister species, depth-mediated speciation may have been involved in their evolution. Additional morphological and genetic analyses of *Coryphopterus* and other goby genera are in progress in efforts to investigate patterns of speciation and historical invasions of deep tropical reefs. Filling gaps in our knowledge of deep-reef species diversity is critical to meaningful hypotheses about the evolution of the deep-reef fauna, and we therefore continue to seek funding for exploratory diving aboard the *Curasub*.

## Supplementary Material

XML Treatment for Coryphopterus curasub

## Appendix

**Appendix:** Links between DNA voucher specimens, GenBank accession numbers, and cytochrome c oxidase subunit I (COI) sequences of *Coryphopterus curasub* sp. n. and *Coryphopterus venezuelae*.

Catalog Number/DNA Number	GenBank No.	GenSeq Designation
*Coryphopterus curasub* sp. n.		
USNM 406373, CUR 11373, Holotype	KT020955	Geneseq-1 COI
USNM 430037, CUR 13302, Paratype	KT020957	Genseq-2 COI
USNM 431328, CUR 14003, Paratype	KT020961	Genseq-2 COI
USNM 430019, CUR 13303, Paratype	KT020958	Genseq-2 COI
*Coryphopterus venezuelae*		
USNM 413804, CUR 12273	KT020959	Genseq-4 COI
USNM 413992, CUR 13008	KT020956	Genseq-4 COI
USNM 430016, CUR 13328	KT020960	Genseq-4 COI
